# Segmented filamentous bacteria are associated with disease activity in children with inflammatory bowel disease

**DOI:** 10.3389/fped.2026.1733148

**Published:** 2026-03-24

**Authors:** Yuling Feng, Huahai Chen, Yeshi Yin, Zhujun Gu, Kai Lin, Weiwei Cheng, Ling Wang, Jing Wang, Jiale Xie, Xiangtao Liu, Wenyan Huang, Haifeng Liu

**Affiliations:** 1 Department of Digestive Endoscopy Center, Shanghai Children’s Hospital, School of Medicine, Shanghai Jiao Tong University, Shanghai, China; 2 Guangxi Key Laboratory of Marine Natural Products and Combinatorial Biosynthesis Chemistry, Institute of Biology, Guangxi Academy of Sciences, Nanning, China; 3 Guangxi Key Laboratory of Animal Reproduction, Breeding and Disease Control, College of Animal Science and Technology, Guangxi University, Nanning, China; 4 Key Laboratory of Comprehensive Utilization of Advantage Plants Resources in Hunan South, College of Chemistry and Bioengineering, Hunan University of Science and Engineering, Yongzhou, Hunan, China; 5 Department of Nephrology, Rheumatology and Immunology, Shanghai Children’s Hospital, School of Medicine, Shanghai Jiao Tong University, Shanghai, China

**Keywords:** children, colonoscopy, inflammatory bowel disease, segmented filamentous bacteria, terminalileum

## Abstract

**Objectives:**

This study aimed to determine the positivity rate of segmented filamentous bacteria (SFB) in pediatric patients with inflammatory bowel disease (IBD) and to evaluate its association with disease activity.

**Methods:**

In this cross-sectional study, 30 pediatric IBD patients and 30 children with functional gastrointestinal disorders were enrolled. SFB in ileal lavage fluid from the terminal ileum were detected using polymerase chain reaction. IBD patients were stratified according to treatment status and disease activity—Untreated Active (UTA), Treated Remission (TRR), Treated Active (TRA)—as well as by disease type (Crohn's disease or ulcerative colitis). SFB positivity rates were compared across subgroups. Within the IBD cohort, disease activity and inflammatory markers, including fecal calprotectin, C-reactive protein, erythrocyte sedimentation rate, platelets count, were compared between SFB-positive and SFB-negative patients.

**Results:**

The SFB positivity rate was significantly higher in pediatric IBD patients than in controls (63.3% vs. 36.7%, *P* = 0.038). Subgroup analysis showed that the UTA group had the highest SFB positivity rate (82.4%), which was significantly higher than both the control group (*P* = 0.002) and the TRR group (28.6%, *P* = 0.021). Among TRA group, the SFB positivity rate remained elevated (50.0%). The positive rate did not differ significantly from that of the UTA group (*P* > 0.05). The SFB positivity rate was higher in pediatric Crohn's disease patients (66.7%, 14/21) than in controls but showed no significant difference compared with ulcerative colitis (*P* > 0.05). SFB-positive patients exhibited significantly higher disease activity indices (PCDAI, *P* = 0.005; PUCAI, *P* = 0.021) and elevated inflammatory markers (fecal calprotectin, *P* = 0.001; C-reactive protein, *P* = 0.001; erythrocyte sedimentation rate, *P* = 0.001) compare with SFB-negative patients.

**Conclusions:**

SFB positivity occurred more frequently in pediatric IBD than in controls—especially in active disease—and was associated with higher inflammatory activity and disease severity.

## 1 Introduction

Inflammatory bowel disease (IBD), comprising Crohn's disease (CD) and ulcerative colitis (UC), is a chronic inflammatory disorder of the gastrointestinal tract. Although its precise etiology remains unclear, IBD is believed to result from a complex interaction among host genetic susceptibility, gut microbiota, and mucosal immune dysregulation (1, 2). The localization, activity, and clinical phenotype of IBD vary markedly among individual, with the mechanisms underlying this heterogeneity still poorly understood. Increasing evidence suggests that interactions between the intestinal immune system and gut microbiota play a pivotal role in IBD pathogenesis (3, 4).

Segmented filamentous bacteria (SFB) are commensal microorganisms with potent immunomodulatory properties, particularly their ability to drive Th17 cell differentiation and influence mucosal immune homeostasis (5, 6). Experimental studies have shown that SFB primarily adheres to the surface of mucosal epithelial cells in the terminal ileum, potentially contributing to the development of autoimmune diseases (7, 8). Although SFB have been identified in the human intestinal (9, 10), their clinical significance and potential involvement in IBD remain largely undefined.

Several animal studies have demonstrated that colonization by SFB is associated with the development or exacerbation of intestinal inflammation and IBD-like pathology (11). Based on these findings, we hypothesized that SFB colonization in the terminal ileum may be associated with intestinal inflammation and disease activity in pediatric IBD. To test this hypothesis, we conducted a cross-sectional study assessing the relationship between SFB positivity and clinical features in pediatric IBD. Ileal lavage samples from the terminal ileum were analyzed by polymerase chain reaction (PCR) to evaluate associations between SFB detection, disease activity, and inflammatory markers.

## 2 Materials and methods

### 2.1 Study population

We conducted a cross-sectional study at Shanghai Children's Hospital, enrolling participants between January 2021 and April 2023. The case group included pediatric patients (≤18 years) diagnosed with CD or UC according to the diagnostic criteria of the European Society for Paediatric Gastroenterology, Hepatology and Nutrition (ESPGHAN) (12). Eligible participants met one of the following conditions: ① newly diagnosed and treatment-naïve patients who had not received any IBD-specific therapy (including immunosuppressants, biologics, or systemic corticosteroids); or ② patients who had completed an 8-week (±7 days) standard induction-remission regimen and returned for scheduled evaluation. The control group consisted of age- and sex- matched children who underwent diagnostic colonoscopy for nonspecific abdominal symptoms (e.g., abdominal pain or bloating) and were ultimately diagnosed with functional gastrointestinal disorders without evidence of organic pathology according to the Rome IV criteria (13). Exclusion criteria were: ① use of antibiotics or probiotics within 1 month prior to enrollment; ② presence of autoimmune disease, immunodeficiency, or primary intestinal malignancy; ③ coexisting chronic gastrointestinal disorders such as celiac disease, eosinophilic gastroenteritis, or infection by specific pathogens; and ④ inability to complete required sample collection or data acquisition.

### 2.2 Grouping and data collection section

To examine the relationship between terminal ileal SFB and inflammatory activity, pediatric IBD patients were categorized using two grouping approaches. First, patients were stratified by treatment status and disease activity into three subgroups: Untreated active (UTA): newly diagnosed patients who had not received any IBD-specific therapy; Treated remission (TRR): patients who achieved clinical remission following standardized induction therapy; Treated active (TRA): patients who remained clinically active despite treatment. Second, patients were classified by disease type as either CD or UC. IBD-specific therapy included immunosuppressants, glucocorticoids, or biologics administered according to established pediatric IBD guidelines.

Disease activity was evaluated using standardized scoring systems: the Pediatric Crohn's Disease Activity Index (PCDAI) for CD and the Pediatric Ulcerative Colitis Activity Index (PUCAI) for UC.

Remission was defined as PCDAI <10 or PUCAI <10.

Additional clinical data collected included age, sex, medication history, endoscopic findings, histopathology reports, and inflammatory markers such as fecal calprotectin (FC), platelet count (PLT), C-reactive protein (CRP), and erythrocyte sedimentation rate (ESR).

### 2.3 Laboratory measurements

FC concentrations were quantitatively measured using an automated fluorescence immunoassay analyzer (FA280, Orienter, Sichuan, China). PLT was determined using an automated hematology analyzer (F-680P, Maccura, Chengdu, China). CRP levels were measured using an automated specific protein analyzer (Ottoman-2020, UPPER, Shanghai, China). ESR was assessed using the Westergren method with an automated ESR analyzer (TEST1, Alifax, Italy). All laboratory measurements were performed in the clinical laboratory of our institution following standardized operating procedures.

### 2.4 Ileal lavage samples collection and SFB extraction

Approximately 6–10 mL of ileal lavage fluid was aseptically collected from the terminal ileum of each patient during endoscopic examination. Samples were transported on ice and processed within 24 h. Each samples were thoroughly mixed using a vortexer. The samples were then allowed to stand for 5 min and centrifuged at 50×*g* for 3 min to precipitate food residues and other large particulate matter. The supernatant was transferred to a clean tube and centrifuged at high speed to obtain bacterial pellet, which were either used immediately or stored at −80 °C.

Bacterial genomic DNA was extracted using a QIAamp DNA Stool Mini Ki (QIAGEN, Hilden, Germany) following the manufacture's protocol. DNA concentrations was quantified using a NanoDrop ND-2000 Spectrophotometer (NanoDrop Technologies, Wilmington, DE, USA), and DNA integrity was confirmed by electrophoresis on 1% agarose gels.

### 2.5 PCR detection of SFB

SFB detection was performed by PCR targeting both the 16S rRNA and flagellin (fliC3/fliC4) gene regions which specifically designed to identify the presence of segmented filamentous bacteria, rather than to characterize overall gut microbiota composition or bacterial genera. The following primers were used:16S rRNA gene primers 779 F(5′-TGTGGGTTGTGAATAACAAT-3′) and 1008 R (5′-GCGGGCTTCCCTCATTACAAGG-3′) (14–16); Flagellin: Flic3/4_F (5′- CAC AAT ATG AAT GCG ATG AAT G-3′) and Flic3/4_R (5′-GCT GTT TGA ATC ATT GAA AT-3′) (17). PCR reactions (25 μL) contained 2.5 μL dNTP (2.5 mmol), 2.5 μL 10 × EX Taq buffer, 1 μL of each primer (10 mmol), 0.3 μL EX Taq polymerase (5 U/μL, TaKaRa), and 2 μL (approximately ∼ 200 ng) template DNA. Thermal cycling consisted of 30 cycles of at 94 °C for 30 s, 56 °C for 30 s, and 72 °C for 45 s, followed by a final extension at 72 °C for 5 min. PCR amplicons were visualized using TAE agarose gel electrophoresis (1.0%) and a gel imager (Bio-Rad, California, USA).

Flagellin PCR products were purified with a Biospin Gel Extraction Kit (Bioer Technology Co., Ltd., Hangzhou, China) and cloned into pMD18-T vectors (Takara, Dalian, China). Ten clones from each library were randomly selected for sequencing using vector- specific M13F primers (Sangon Biotech, Shanghai, China). This process yielded 10 high-quality flagellin sequences from each library. The presence of SFB-specific sequences was considered PCR-positive (10, 18). Samples were classified as SFB-positive if either the 16S rRNA PCR assay or the flagellin PCR assay yielded a positive result.

### 2.6 Statistics

The SPSS software package (version 22.0; IBM SPSS Statistics for MacBook, Armonk, NY, USA) was used for all statistical analyses. Continuous variables were expressed as mean ± SD or median (IQR), depending on their distribution. Categorical variables were compared using the chi-square or Fisher's exact test, and continuous variables using the *t*-test or Mann–Whiney *U* test, as appropriate. A two-tailed *P* < 0.05 was considered statistical significant.

## 3 Results

### 3.1 Baseline characteristics

A total of 60 subjects were enrolled, including 30 children with IBD and 30 controls. Among the IBD case group, 21 (70%) patients were diagnosed with CD and 9 (30%) with UC. The group was further subdivided into UTA (*n* = 17), TRR (*n* = 7), and TRA (*n* = 6). Baseline demographic analysis showed no significant differences in age and sex distribution between the IBD and control groups or among IBD subgroups (*P* > 0.05). Similarly, the proportions of CD and UC did not differ significantly across the three IBD subgroups (*P* > 0.05). As summarized in Table 1, inflammatory indicators including FC, CRP, and ESR differed significantly among the study groups (*P* < 0.05), whereas PLT did not (*P* > 0.05).

**Table 1:** Baseline characteristics of study participants.

Characteristic	C, *n* = 30	IBD, *n* = 30	UTA, *n* = 17	TRR, *n* = 7	TRA, *n* = 6	*P*-value (overall)	*P*-value (C vs. IBD)
Demographics							
Age, years, median (IQR)	10.25 (7.5–14)	12.54 (7.5–13.94)	12.5 (5.04–13.84)	12.92 (6–15.75)	11.42 (8.73–13.94)	0.784	0.950
Male, *n* (%)	19 (63.33)	19 (63.33)	12 (70.59)	4 (57.14)	3 (50)	0.810	>0.999
Disease classification							
CD, *n* (%)	/	21 (70)	10 (58.82)	6 (85.71)	5 (83.33)	0.310	/
UC, *n* (%)	/	9 (30)	7 (11.18)	1 (14.29)	1 (16.67)	/	/
Inflammatory markers							
Fecal calprotectin, ug/g, median (IQR)	7.5 (5, 30)	43.15 (30, 174.7)	53.62 (35.19, 200.1)	30 (30, 30.32)	82.51 (39.3, 262.3)	<0.001	<0.001
CRP, mg/L, median (IQR)	3 (1, 6)	5 (5, 10.75)	8 (5, 26)	5 (3, 5）	5 (2.25, 6.25)	0.007	0.007
PLT, ×10^9^/L, median (IQR)	307.5 (235.5, 365)	308.5 (282, 453.8)	307 (285, 518.5)	310 (245, 374)	319 (239.5, 401.5)	0.715	0.368
ESR, mm/h, median (IQR)	3 (2, 9.25)	20 (9.75, 46.25)	25 (7, 60.5)	12 (9, 25)	21.5 (13.75, 35.25)	<0.001	<0.001

C, control; IBD, inflammatory bowel disease; UTA, untreated active; TRR, treated remission; TRA, treated active.

*P*-value for the overall comparison across the four groups (C, UTA, TRR, TRA) by Fisher's exact test.

### 3.2 SFB detection and distribution

The overall SFB PCR positivity rate was significantly higher in IBD than in controls (63.3% vs. 33.3%, *P* = 0.038).

**Figure 1:**
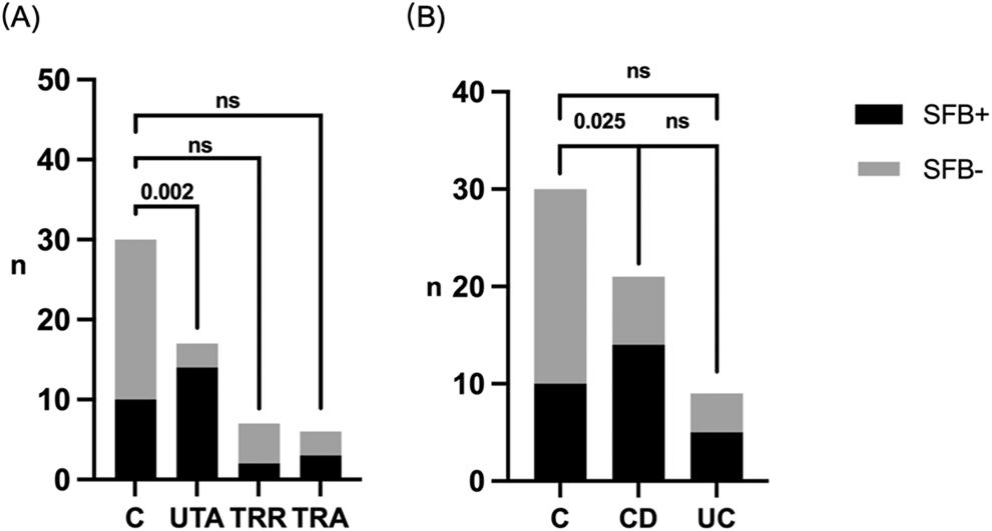
Detection and distribution of segmented filamentous bacteria (SFB). **(A)** Comparison of SFB positivity between the control group and each IBD subgroup. The UTA group showed a significantly higher SFB positivity rate than the control group, whereas differences among other subgroups were not significant. **(B)** Comparison of SFB positivity among the control, CD, and UC. SFB positivity was significantly higher in the CD compared with controls, while no significant difference was observed among other groups. Statistical analysis was performed using Fisher's exact test (NS = *P* > 0.05). UTA, untreated active; TRR, treated remission; TRA, treated active; CD, Crohn's disease; UC, ulcerative colitis.

When stratified by disease activity and treatment status, SFB positivity was highest in the UTA subgroup (82.4%), significantly exceeding that of the control group (33.3%, *P* = 0.002) and the TRR subgroup (28.6%, *P* = 0.021; Figure 1A). The positivity rate in the TRA subgroup remained elevated (50.0%) and did not differ significantly from the UTA group (*P* = 0.279).

Analysis by disease types showed a higher SFB positivity rate in CD patients compared with controls (66.7% vs. 33.3%, *P* = 0.025). A similar trend was observed in UC (55.6%), although the difference was not statistically significant (*P* = 0.266). The positivity rate did not differ between CD and UC (*P* = 0.687; Figure 1B).

### 3.3 Association between SFB and disease activity

**Figure 2:**
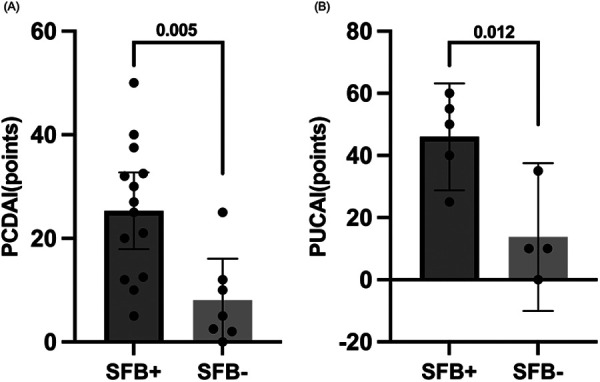
Association between SFB positivity and disease severity in pediatric IBD. **(A)** Pediatric Crohn's Disease Activity Index (PCDAI) scores were significantly higher in SFB-positive than in SFB-negative children. **(B)** Pediatric Ulcerative Colitis Activity Index (PUCAI) scores were also significantly higher in SFB-positive patients. Statistical significance was assessed using Student’ s *t*-test.

Among IBD patients, SFB-positive children exhibited significantly higher disease activity index scores than SFB-negative children (Figure 2). Specifically, SFB-positive CD patients had higher PCDAI scores (*P* = 0.005), and SFB-positive UC patients had higher PUCAI scores (*P* = 0.012).

### 3.4 Correlation between SFB and inflammatory markers

**Figure 3:**
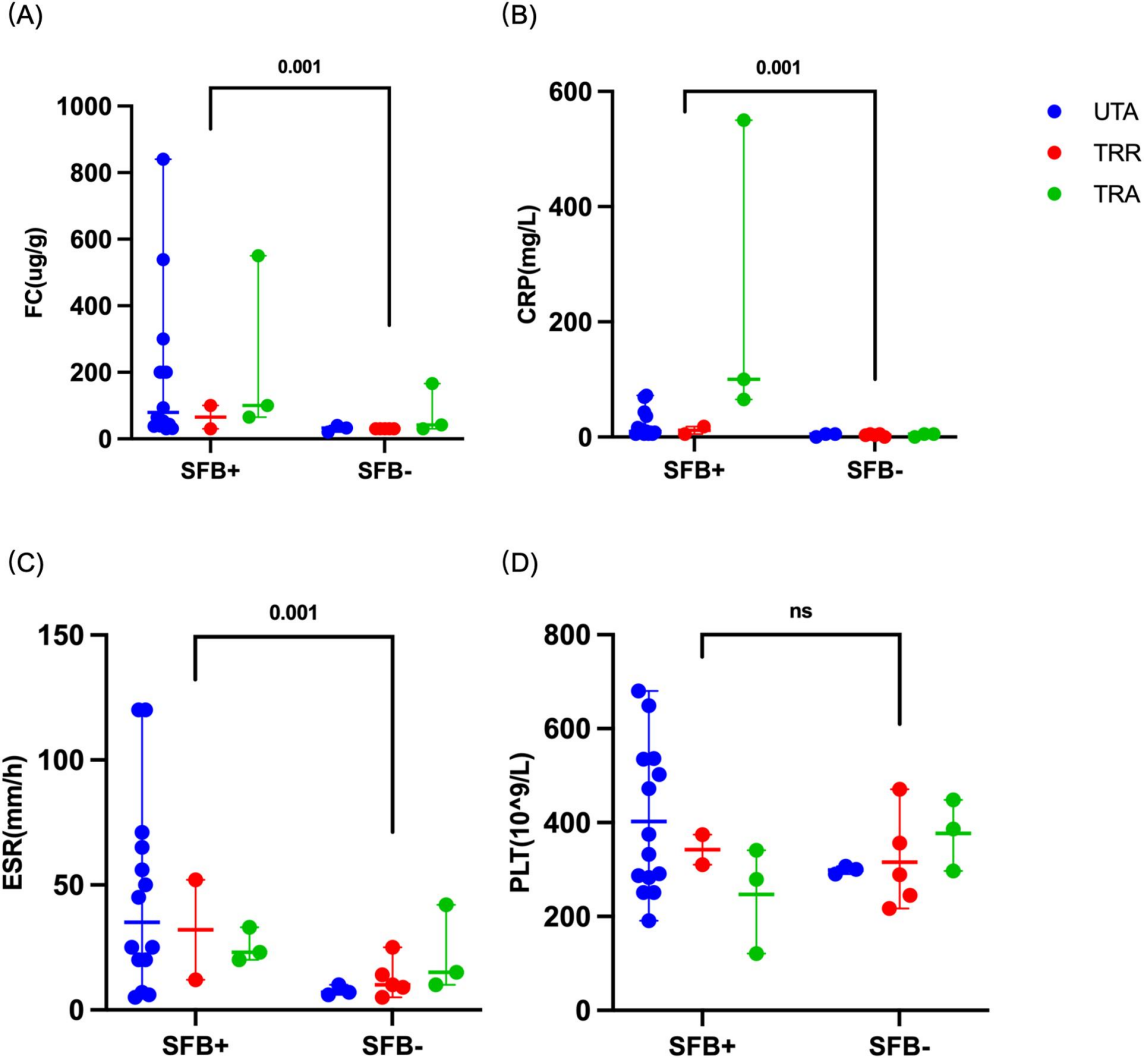
Comparison of inflammatory biomarkers between SFB-positive and SFB-negative patients. **(A–C)** Fecal calprotectin (FC), C-reactive protein (CRP), and erythrocyte sedimentation rate (ESR) levels were significantly higher in SFB-positive patients than in SFB-negative patients. **(D)** Platelet (PLT) counts did not differ significantly between the two groups. Statistical significance was assessed using the Mann–Whitney *U* test for FC, CRP, and ESR, and Student's *t*-test for PLT (NS = *P* > 0.05). UTA, untreated active; TRR, treated remission; TRA, treated active.

To further assess the potential relationship between SFB colonization and intestinal inflammation, inflammatory markers were compare between SFB-positive and SFB-negative IBD patients. SFB-positive children had significantly higher levels of FC (*P* = 0.001), CRP (*P* = 0.001), and ESR (*P* = 0.001) than SFB-negative children. While PLT level did not differ significantly (*P* = 0.384; Figure 3).

## 4 Discussion

This cross-sectional study demonstrates that the presence of SFB in the terminal ileum was associated with increased disease activity and inflammatory burden in pediatric IBD. SFB was more freguently detected in children with IBD than in controls, with a significantly higher prevalence in CD. A similar upward trend was observed in UC patients, although this difference did not reach statistical significance. Within the IBD cohort, SFB positivity was highest in untreated patients with active disease and was associated with a more severe inflammatory phenotype.

The higher SFB positivity observed in pediatric IBD is consistent with previous studies reporting characteristic alterations in the gut microbiota of IBD patients (3, 18–21). Caselli et al. reported histological identification of SFB-like organisms in resected ileo-cecal tissue specimens from patients with UC, with lower detection rates in controls, providing morphological evidence of SFB presence in human intestinal tissue (22). Although the distribution pattern reported by Caselli et al. differs from that observed in our cohort, such discrepancies may reflect differences in patient age, sampling methods and detection techniques. This association identified in CD compared with UC may reflect the pathophysiological features of the two diseases. CD is characterized by transmural inflammation that can effect any segment of the gastrointestinal tract, most commonly the terminal ileum—also the principal site of SFB colonization (7, 10, 23). In contrast, UC is limited to the colonic mucosa. The anatomical overlap supports the hypothesis that SFB may be more specific to lesions in the terminal ileum. Experimental research has shown that SFB attach to small intestinal epithelial cells, modulate T-cell differentiation, and elicit robust mucosal immune responses, thereby influencing local inflammatory processes (4–7).

SFB positivity varied across clinical subgroups of IBD. The highest positivity rate observed in untreated active patients, followed by patients with persistent disease activity after treatment, whereas patients in remission had the lowest rate (Figure 1A). These findings suggest a potential association between SFB positivity and IBD disease activity. The similar positivity rates observed in untreated and treated active patients may reflect a direction that merits further study. This suggests SFB positivity may complement existing inflammatory biomarkers. Moreover, within the IBD cohort, SFB-positive patients patients had higher disease activity index and elevated inflammatory markers (FC, CRP, and ESR; Figure 3). The correlation between SFB detection and inflammatory markers reinforces its potential role in amplifying intestinal inflammation or sustaining disease progression. This clinical observation aligns with prior animal studies demonstrating that SFB can promote autoimmune and inflammatory conditions through modulation of intestinal microbiota, alteration of mucosal immune homeostasis, and induction of Th17-mediated immune responses (5–7, 24). SFB abundance is positively correlated with the severity of Crohn's disease-like ileitis (11). Therefore, We speculate that in our pediatric patients, SFB may amplify intestinal inflammation through a similar Th17-mediated pathway, creating a microenvironment that perpetuates disease activity and potentially diminishes the efficacy of conventional therapies.

Despite these findings, several limitations must be acknowledged. First, the relatively small sample size may limit statistical power and the stability of effect estimates; therefore, the findings should be interpreted with caution. Second, due to ethical constraints and the invasive nature of ileal endoscopy, healthy children could not be included as controls; instead, children with functional gastrointestinal disorders according to the Rome IV criteria were used as a pragmatic comparison group, which may not represent a microbiologically neutral baseline. Third, SFB detection relied on targeted PCR-based identification without quantification of bacterial load, precluding assessment of dose–response relationships and limiting the ability to distinguish between causative, permissive, or secondary colonization roles of SFB. Finally, the cross-sectional design of this study does not allow causal inference, and longitudinal, multicenter studies incorporating quantitative microbial and immunological analyses are required to validate and extend these findings.

In conclusion, our findings suggest that the SFB colonization in the terminal ileum is closely associated with active disease and heightened inflammatory activity in pediatric IBD. Although causality cannot yet be established, SFB may represent microbial factor involved in intestinal immune dysregulation and could serve as a promising focus for future mechanistic and therapeutic research in pediatric IBD.

## Data Availability

The original contributions presented in the study are included in the article/Supplementary Material, further inquiries can be directed to the corresponding author.
