# Supplementary material for: Segmented filamentous bacteria are associated with disease activity in children with inflammatory bowel disease
Source: Front Pediatr. 2026 Mar 24;14:1733148. doi: 10.3389/fped.2026.1733148 (PMC13055543; doi:10.3389/fped.2026.1733148)
Supplement: Supplementary file 1 [file Datasheet1.pdf]

Supplementary Material

1 Supplementary Tables

Supplementary Table 1. Odds ratios (ORs) with 95% confidence intervals for SFB positivity across clinical subgroups

Comparison	OR	95% CI	P value	Statistical test
C vs. UTA	0.11	0.03-0.42	0.002	Fisher's exact test
C vs. TRR	1.25	0.19-7.11	> 0.99	Fisher's exact test
C vs. TRA	050	0.10-2.50	0.65	Fisher's exact test
TRR vs. TRA	0.40	0.05-3.16	0.592	Fisher's exact test
UTA vs. TRR	11.67	1.70-70.55	0.021	Fisher's exact test
UTA+TRA vs. TRR	3.27	0.51-17.91	0.232	Fisher's exact test
UTA vs. TRA	4.67	073-26.94	0.279	Fisher's exact test
C vs. IBD	0.29	0.09-0.88	0.038	Fisher's exact test
C vs. CD	0.25	0.07-0.82	0.025	Fisher's exact test
C vs. UC	2.50	0.53-9.39	0.266	Fisher's exact test
CD vs. UC	1.60	0.39-7.42	0.687	Fisher's exact test

Abbreviations: UTA, untreated active; TRR, treated remission; TRA, treated active; IBD, inflammatory bowel disease; CD, Crohn's disease; UC, ulcerative colitis.
